# Are Cesarean Section and Appendectomy in Pregnancy and Puerperium Interrelated? A Cohort Study

**DOI:** 10.3389/fsurg.2022.819418

**Published:** 2022-02-17

**Authors:** Banuhan Şahin, Andrea Tinelli, Goran Augustin

**Affiliations:** ^1^Gynecology and Obstetrics Department, Amasya University Sabuncuoglu Serefeddin Training and Research Hospital, Amasya, Turkey; ^2^Department of Obstetrics and Gynecology and CERICSAL (Centro di RIcerca Clinica SALentino), “Veris Delli Ponti Hospital”, Lecce, Italy; ^3^Division of Experimental Endoscopic Surgery, Imaging, Technology and Minimally Invasive Therapy, Vito Fazzi Hospital, Lecce, Italy; ^4^Laboratory of Human Physiology, Faculty of Biological and Medical Physics, Phystech BioMed School, Moscow Institute of Physics and Technology (State University), Moscow Region, Russia; ^5^School of Medicine University of Zagreb, Zagreb, Croatia; ^6^Department of Surgery, University Hospital Centre Zagreb, Zagreb, Croatia

**Keywords:** appendectomy, Cesarean section, delivery, postpartum, pregnancy complications, puerperium

## Abstract

**Introduction:**

It is not known whether appendectomy for acute appendicitis (AA) increases the Cesarean section (CS) rate and whether CS increases the likelihood of AA and appendectomy in the early puerperium. In this study, delivery type and delivery outcomes and appendectomy during pregnancy and puerperium were analyzed.

**Methods:**

This cross-sectional retrospective study was performed on 11,513 women, delivered during 2015–2020. Inclusion criteria were patients undergoing appendectomy for AA during pregnancy and the first 6 weeks after delivery. Evaluating parameters were age, parity, gestational week at birth, delivery type, and babies' birth weight.

**Results:**

Thirty-two patients underwent appendectomy: 12 during pregnancy (2 in the first trimester, 6 in the second trimester, 4 in the third trimester) and 20 women during puerperium. 58.2% of pregnant women and 65% of puerperal women were submitted to CS.

**Discussion:**

Half of the women who underwent appendectomy for AA during pregnancy may require urgent CS. The cause of acute abdomen in the postpartum period, especially in the first week, could be AA, especially in women delivered by CS.

## Introduction

Acute appendicitis (AA) is the most common cause of non-obstetric acute abdominal pain during pregnancy (1). It occurs in 1/2,000 to 1/500 pregnancies (2, 3), and it is exceedingly rare in the third trimester, possibly due to the protective effects of hormonal and immunological changes during pregnancy (4–6). The diagnosis during pregnancy is challenging due to either nonspecific signs and symptoms or inconclusive laboratory test results due to physiological changes or limited options for radiological imaging (7). During pregnancy, if not (partly) fixed or retroperitoneal, the appendix is slightly displaced upward due to the growing uterus, especially in the second and third trimesters. Still, it returns to its previous location just 10 days after birth (8–10).

AA is the most common non-urogenital puerperal condition and the most frequent cause for hospital readmission in the puerperium (11). Postpartum diagnosis of AA is challenging due to atypical presentation and broad differential diagnosis, including urinary infections, pneumonia, cholecystitis, and many gynecological infections, such as puerperal endometritis and tubo-ovarian abscess (12). However, perforated AA is a frequent cause of postpartum sepsis, second only to puerperal sepsis (13).

Many studies have shown that AA during pregnancy can result in preterm delivery, while fetal loss is significant after perforated AA (14–16). However, due to the low number of cases in all these studies, the significance of the relationship between appendectomy for AA and pregnancy complications and delivery outcomes is misinterpreted (17). Also, the relationships between appendectomy for AA in puerperium and the routes of previous delivery are not previously reported. In this study, the authors evaluated the association between appendectomy for AA during pregnancy and puerperium and birth outcomes.

## Methods

A retrospective cross-sectional study included the analysis of the hospital records of pregnant women who attended prenatal follow-up and delivered from October 1, 2015, to October 1, 2020. Ethical approval for the study was granted by the Local Institutional Review Board with decision no: 2020/9–98. This study was registered in clinicaltrials.gov: NCT04876547 https://clinicaltrials.gov/ct2/show/NCT04876547.

Women aged 18–45 years undergoing appendectomy for AA during pregnancy or within the first 6 weeks after delivery were included. Cases with missing data were excluded. Analyzed parameters were demographic data, prenatal follow-up, birth results (live birth/stillbirth), a gestational week at birth, delivery type, birth weight of babies, postpartum records (post-partum course, early and late complications), the timing of appendectomy (prenatal vs. postpartum), operation notes (description of the intervention and intra- and post-operative complications), and pathology reports. The following parameters in women who had an appendectomy during pregnancy and puerperium within six postpartum weeks were compared by age, a gestational week at birth, birth weight, and the delivery types. Also, pregnancy complications that could have been associated with appendectomy (abortion, preterm birth, stillbirth, etc.) were recorded. The only indication of an urgent Cesarean section (CS) in such patients is an infectious condition. A pregnant woman with fever is at risk for severe complications and needs urgent treatment (also with urgent CS). In that case, CS was performed due to fetal distress without uterine contractions and any other potential cause was found. Authors excluded other non-medical indications for CS, such as CS on patient's request, and medical indications such as dystocia or previous CS.

### Statistical Analysis

The study data were analyzed statistically using the IBM SPSS Statistics version 21.0 software (IBM Corp., Armonk, NY). Descriptive statistics (mean, median, standard deviation, minimum, maximum, number, percentage) were used to depict data. The Chi-square Test and The Mann-Whitney U test compared the two groups (since parametric assumptions were not met). The Kruskal Wallis Test compared the three groups (since parametric assumptions were not met). Comparisons yielding *p*-values of < 0.05 were defined to be statistically significant.

## Results

A total of 11,513 singleton pregnant women were evaluated, 6,531 women delivered vaginally, and 4,982 women delivered by CS. Thirty-two patients underwent appendectomy: 12 women during their pregnancies and 20 women during puerperium. The mean age of pregnant women who had appendectomy for AA was 31.5 ± 5.5 years. Five of these 12 pregnant women were in their first pregnancy. Two pregnant women have been undergoing appendectomy in the first trimester, 6 in the second trimester, and 4 in the last trimester of pregnancy. One pregnancy resulted in a stillbirth at the 22^nd^ week of gestation, with signs of perforated AA and intra-abdominal sepsis. Other pregnancies treated by appendectomy resulted in live births, but two women in their second trimester and four women in their last trimester had a preterm delivery (Table 1). The mean week of gestation of 12 pregnant women at the time of appendectomy was 24.7 ± 9.5 weeks; with mean gestational age at birth was 35.5 ± 5.2 weeks, according to trimesters: 39 ± 1.4 weeks, 33.6 ± 7.0 weeks, and 36.7 ± 1.5 weeks, respectively. There was no statistical difference between the groups (p = 0.421). The mean birth weight was 2,928 ± 871 grams, according to trimesters: 3,667 ± 300 grams, 2,625 ± 1,083 grams, 3,015 ± 491 grams, respectively. There was no statistical difference between the groups (*p* = 0.217). It was observed that five pregnant women had a vaginal birth, and seven pregnant women had CS. The mean gestational age at live birth of the healthy pregnant cohort was 38.4 ± 4.2 weeks, and the mean birth weight was 3,091 ± 1,458 grams. The difference between healthy pregnant women and pregnant women with appendectomy for the birth week and birth weight could not reach a statistical value (*p* > 0.05 and *p* > 0.05).

**Table 1:** Characteristics of pregnant women who underwent an appendectomy.

**Case**	**Age**	**Appendectomy**	**Delivery**	**Delivery**	**Birth**
	**(year)**	**week**	**week**	**route**	**weight (gr)**
1	38	22	22	Vaginal	550
2	29	24	38	CS	3,000
3	30	28	39	CS	3,430
4	36	22	29	vaginal	2,570
5	27	8	38	CS	3,455
6	33	34	36	CS	2,500
7	28	12	40	vaginal	3,880
8	28	21	34	vaginal	2,700
9	29	36	36	CS	3,000
10	45	18	40	vaginal	3,500
11	26	36	36	CS	2,880
12	30	36	39	CS	3,680

*CS, Cesarean section*.

All of 20 patients operated by appendectomy in the puerperium had a singleton pregnancy which had resulted in a live birth. The average age of women who had undergone appendectomy for AA in the puerperium was 29.7 ± 5.8 years. Twelve women on 20 were multiparous. Seven puerperal women had a vaginal birth, and 13 puerperal women had CS. The mean postpartum appendectomy week was 3 ± 2.05. Eight women (40% of all puerperal appendectomies) have been operated on during the first postpartum week. Two of them had a vaginal birth, and six had CS. Two cases (25% of women who had undergone appendectomy during the first week) operated on during the first postpartum week were diagnosed with perforated AA. The mean gestational age at birth was 37.1 ± 5.7 weeks and the mean birth weight was 3,296 ± 592 grams. There was no difference between healthy puerperal women and puerperal women with appendectomy for the birth week and birth weight (*p* > 0.05 and *p* > 0.05).

Most women who had an appendectomy for AA during pregnancy had an urgent CS (Table 2). Also, most women who had an appendectomy during puerperium had a CS (58 vs. 65%, *p* = 0.706). For comparison, the CS rate of the entire healthy pregnant population was 43.27%.

**Table 2:** Characteristics of women who underwent appendectomy during pregnancy and puerperium.

	**Pregnancy**	**Postpartum**	***P*** **value**
	**(*n* = 12)**	**(*n* = 20)**	
Age (years)	31.5 ± 5.5	29.7 ± 5.8	0.458
Proportion of primipara	5 (41.7)	8 (40)	0.926
Gestational week at delivery	35.5 ± 5.2	37.1 ± 5.7	0.152
**Delivery route**			
Vaginal	5 (41.7)	7 (35)	0.706
Cesarean section	7 (58.3)	13 (65)	
Newborn weight (gr)	2,928 ± 871	3,296 ± 592	0.227
**Cesarean indications**			
Fetal distress	7 (100)	2 (15.3)	0.182
Dystocia	0	8 (61.5)	
Breech position	0	3 (23.0)	

*Variables presented as mean ± sd and n (%)*.

## Discussion

This is the first study analyzing specifically the relationship between the timing of appendectomy and the type of delivery. It was observed that pregnant women who underwent appendectomy during pregnancy, especially in the second trimester, gave preterm birth with smaller newborns. On the other hand, when AA occurs in the third trimester, prematurity is significantly reduced. One of the causes could be that during the second trimester, the woman reduces the caloric intake due to maternal illness with inflammation and stress from the operation due to AA. Only one operated patient in pregnancy developed sepsis after appendiceal perforation due to the inability to diagnose, resulting in the death of the fetus at the 22^nd^ week of gestation, while six women had preterm delivery. The first important finding is that most women who had undergone appendectomy (in both groups) were delivered by CS. In our series, two pregnant women underwent concurrent appendectomy with urgent CS in the 36 [th] gestational week and one pregnant woman underwent an appendectomy in her 34^th^ gestational week and then had an urgent CS after two weeks later due to fetal distress related to persistent infection.

About 40% of postpartum women had undergone appendectomy within the first postpartum week, and 2/8 had perforative AA. Puerperal women may associate their complaints, including localized pain, with the recent CS. Also, analgesics can mask or diminish the severity of clinical presentation. Therefore, these factors could partly explain the delay in diagnosis and treatment and the high incidence of perforated AA in this monitored group.

The second interesting finding is that puerperal appendectomy was frequent after a CS (65%). Most appendectomies were during the first week after CS (75%). Concerning this evidence, any foci of infection that may develop due to a lack of compliance with asepsis during CS (open abdominal operation) and lack of preoperative antibiotic therapy or invasion of the amnion fluid may lead to AA (18). It is difficult to conclude the cause of AA. It could be AA (which starts from the appendiceal mucosa) or periappendicitis when a surrounding infection spreads to the appendix (19). Another potential cause of post-CS AA could be the change in the location of the appendix during intraoperative uterine manipulation. If the appendix is partly fixed, manipulation can cause kinking producing partial or complete obstruction of the appendiceal lumen finally resulting in AA.

The first limitation is a relatively small number of cases for firm conclusions. There should be worldwide multicentric studies with more patients to deliver firm conclusions. Although 11513 pregnancies were scanned in the last 5 years from hospital records, the number of appendectomies performed was only 32 overall. In our clinic, the rate of CS in the last 5 years has been 43.2%. Since our hospital is a referral hospital for the surrounding districts, many pregnant women with complicated pregnancies have been referred for treatment. Another limitation could be the indications for CS which vary widely around the globe. Also, specific indications for CS and their relationship to postpartum appendicitis/appendectomy in multicentric studies could deliver firm conclusions.

In conclusion, Appendectomy for nonperforated AA in pregnancy does not increase severe fetal morbidities, except for the possibility of urgent CS with all its consequences. However, considering the number of surgeries, the authors' opinion is that the protective effect of pregnancy on AA immediately ends in the early postpartum period. Therefore, it should be kept in mind that the cause of acute abdomen in the first six postpartum weeks, especially during the first week, could be AA. The incidence of AA is additionally increased in women delivered by CS, indicating that appendectomy should be made early in such cases.

## Data Availability Statement

The raw data supporting the conclusions of this article will be made available by the authors, without undue reservation.

## Ethics Statement

The studies involving human participants were reviewed and approved by Amasya University local Ethics Committee with no: 2020/9-98. The patients/participants provided their written informed consent to participate in this study. Written informed consent was obtained from the individuals for the publication of any potentially identifiable images or data included in this article.

## Disclosure

The authors alone are responsible for the content and writing of the article.

## Author Contributions

BŞ contributed to data collection, analysis, and manuscript writing. AT contributed to study design, manuscript writing, and revision. GA contributed to manuscript writing and revision. All authors approved the final version of the article for submission. All authors contributed to the article and approved the submitted version.

## Conflict of Interest

The authors declare that the research was conducted in the absence of any commercial or financial relationships that could be construed as a potential conflict of interest.

## Publisher's Note

All claims expressed in this article are solely those of the authors and do not necessarily represent those of their affiliated organizations, or those of the publisher, the editors and the reviewers. Any product that may be evaluated in this article, or claim that may be made by its manufacturer, is not guaranteed or endorsed by the publisher.
